# Pennsylvania State Veterinary Medical Association

**Published:** 1890-04

**Authors:** S. J. J. Harger

**Affiliations:** Secretary


*Pennsylvania State Veterinary Medical Association.*—The annual meeting of the Pennsylvania State Veterinary Medical Association was held on March 4th, 1890, at the Veterinary Department of the University of Pennsylvania, Philadelphia. The meeting was called to order at 10 A. M. by the president, Dr. W. L. Zuill.

The following members responded to roll-call: W. S. Kooker, J. R. Hart, C. Schauffler, M. J. Collins, J. C. Michener, P. M. Minster, S. E. Weber, W. H. Knight, W. N. Custer, H. W. George, Z. S. Keil, W. H. Hoskins, R. Gladfelter, R. S. Huidekoper, W. L. Zuill, C. Goentner, N. Rechtenwald, I. Michener, R. G. Webster, W. H. Ridge, D. C. Stanton, George Magee, F. B. Raynor, J. B. Raynor, G. B. Raynor, F. Bridge, W. S. Phillips, C. Williams, Robert Formad, N. E. Reinhart and S. J. J. Harger.

Dr. W. E. B. Miller was sent as a delegate by the New Jersey State Association. The association was also honored by the presence of Drs. J. A. Marshall, James McCourt, Martine, H. Raynor.

The minutes of the last meeting were read and approved. The election of officers for the ensuing year resulted as follows: President, W. S. Kooker; first vice-president, T. B. Raynor; second vice-president, J. C. Michener; third vice-president, N. Rechtenwald; recording secretary, S. J. J. Harger; corresponding secretary, R. Gladfelter; treasurer, J. R. Hart; board of trustees, W. H. Hoskins, chairman; R. S. Huidekoper, J. C. Michener, S. E. Weber, Charles Goentner. Twelve applications for membership were received. A recess of one-half hour was taken to examine the applicants.

Drs. F. F. Hoffman, H. S. Borneman, P. H. Seltzer, J. Helmar, J. H. Tinberman, C. H. Magill, H. A. Paget, J. B. Irons, J. H. Oyler, L. O. Lusson, L. E. Wheat and A. Diemer were elected members. Drs. Koons, Bradley and Kilbride were absent, and the names of Drs. W. B. Werntz and J. P. Vasey were laid over until next meeting. Dr. Hoskins, chairman of the Committee on Legislation, made a report stating that litigation is being carried on in five counties. Injunctions have been served against post-registrations in Lancaster County. The registrations in Pennsylvania are as follows: Graduates, 157; existing practitioners, 752; illegal, 136; post-registrations, 82. Dr. Kooker, secretary of the same committee, read a large number of communications. The committee on "Intelligence and Education," made no report. Dr. Bridge, chairman of the Committee on Contagious Diseases and Sanitary Police, reported verbally that glanders, tuberculosis, chicken-cholera and hog-cholera are prevalent in the State. He recommended a State appropriation to indemnify the owners of such animals as should be destroyed.

A communication from S. S. Moyer, stating his withdrawal from the association, was read and laid on the table.

Dr. Huidekoper addressed the meeting on "Contraction of the Feet and Contracted Heels." He reviewed the studies on the elasticity of normal and diseased feet, and laid special stress upon the diseases of bones and tendons in distal parts of the legs which may be caused by contraction, as well as the troubles inside of the horny covering, which latter are generally due to atrophy, followed or not by inflammation.

Dr. Isaiah Michener then addressed the meeting on the question of cribbing as an unsoundness. He called attention to adverse decisions in the courts of law, which have declared it to be a disease, and again have declared it to be only a habit. After defining the modes of deglutition of air and the epiphenomena of indigestion, he classed it primarily as a nervous disorder, complicated by digestive diseases. He cited cases in his own practice of heredity. A stallion, which was a cribber, got progeny, which all became cribbers; a cribbing mare had a foal which became a cribber before it was six months old.

Dr. W. H. Ridge read a very able paper on "Parturient Apoplexy." He claims that no benefit is derived from purgatives. His treatment consists principally of small and repeated doses of aconite and belladonna. With this treatment his mortality has been only about 50 per centum. Dr. Hoskins offered a resolution endorsing the Army Veterinary bill of the United States Veterinary Medical Association, now before Congress. Dr. Hoskins moved that a delegation of three from this association attend the meeting of the New Jersey State Veterinary Medical Association.

An invitation was extended to the association to attend the next meeting of the United States Veterinary Medical Association, on September 16th, in Chicago.

The President appointed the following committees: Intelligence and Education, R. S. Huidekoper, chairman; J. W. Sallada and R. A. Paget. Sanitary Police, W. L. Zuill, chairman; F. Bridge and J. B. Irons. Legislation, W. H. Hoskins, chairman; R. S. Huidekoper, F. Bridge, James B. Raynor and Robert Gladfelter. Delegates to the New Jersey State Veterinary Association, W. H. Hoskins, C. H. Magill, P. M. Minster.

The essayists for next meeting will be Alexander Glass, S. E. Weber, W. L. Zuill.

The next semi-annual meeting will be held at Lancaster in September.

S. J. J. Harger, *Secretary.*

